# Supplementary material for: A Conserved Landscape of Chemokine Receptor Co-expression Defines the Functional States of CD8+ T Cells in Melanoma
Source: bioRxiv. 2025 Dec 16:2025.12.10.693486. Preprint. [Version 2] doi: 10.64898/2025.12.10.693486 (PMC12710859; doi:10.64898/2025.12.10.693486)
Supplement: Supplement 1 [file media-1.pdf]

Supplementary Figure 1.

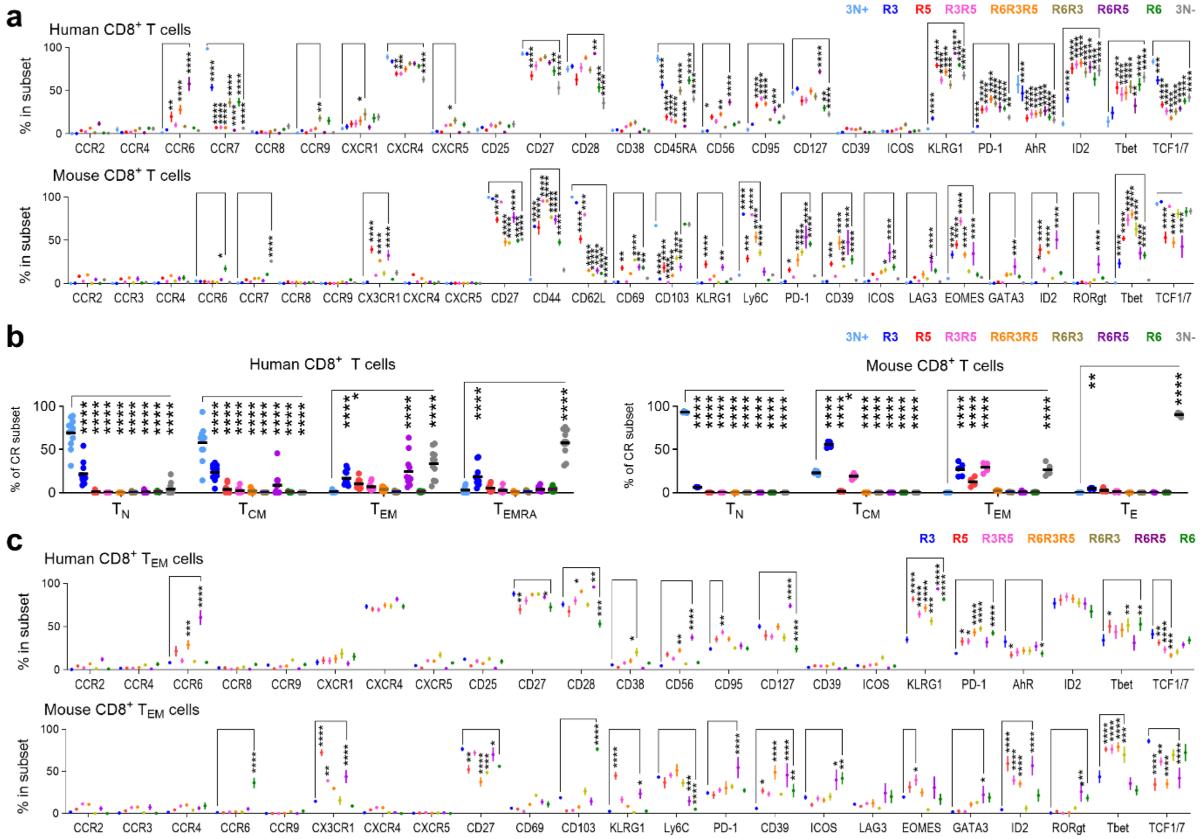

Supplementary Figure 1. Phenotypic and functional characterization of CR-defined T cell subsets. a. Expression of CRs, transcription factors, activation and exhaustion markers across CR-defined $CD8^+$ T-cell subsets (corresponding to Fig. 2d). **b**. Distribution of CR-defined subsets within T-cell memory subsets (corresponding to Fig. 2e). **c**. Expression of CRs, transcription factors, activation and exhaustion markers among CR-defined $CD8^+$ T_{EM} cell subsets (corresponding to Fig. 2f). Statistical significance determined by unpaired Mann-Whitney tests, *P < 0.05, **P < 0.01, ***P < 0.001, ****P < 0.0001.

Supplementary Figure 2.

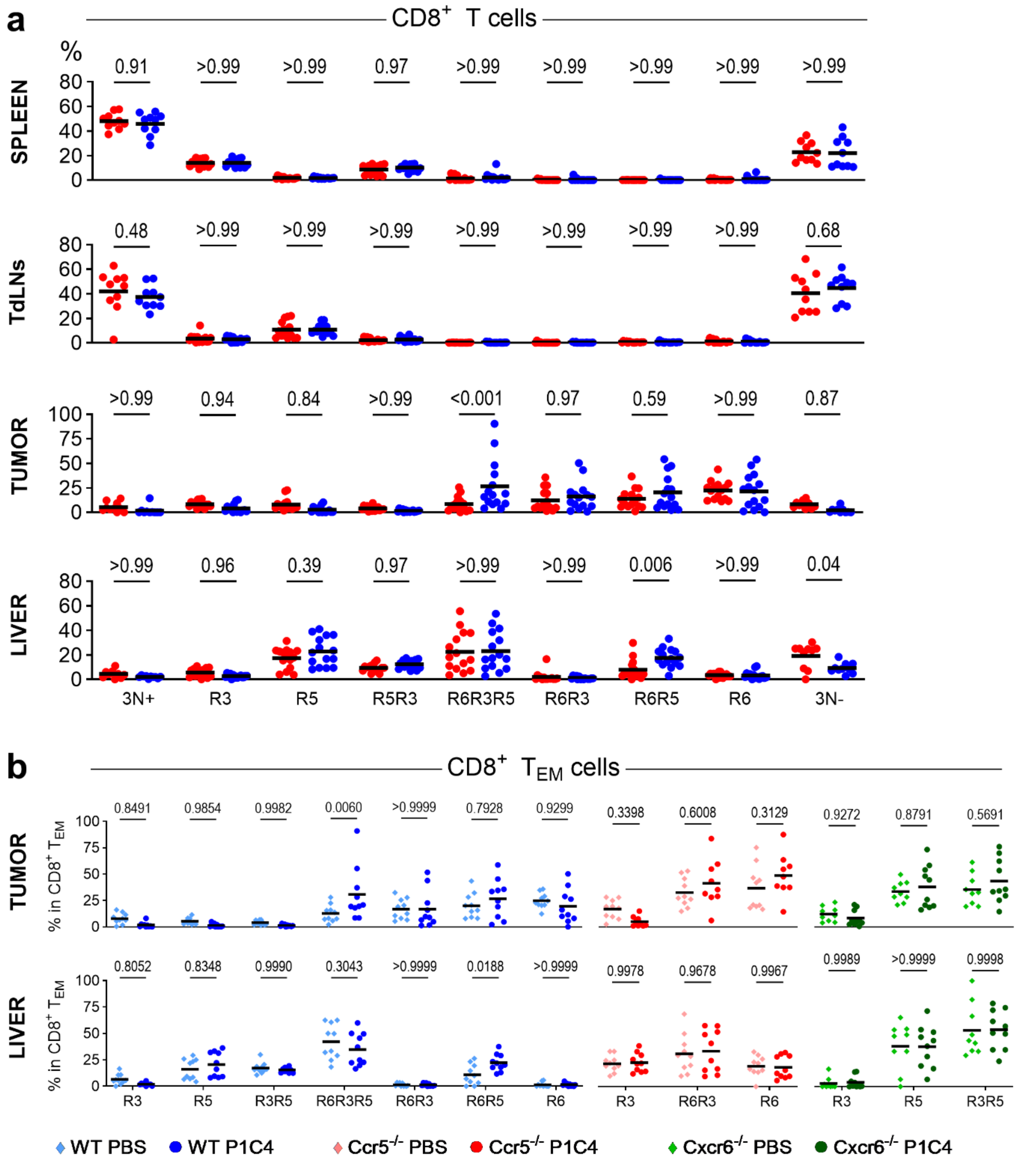

Supplementary Figure 2. Tissue-specific modulation of CR-defined subsets by immune checkpoint blockade. a. Percentages of CR-defined subsets in spleen, tumor-draining lymph nodes (TdLNs), tumor and liver following P1C4 treatment compared to PBS control group (expanded data for Fig. 3c and Extended Data Fig. 5f). **b**. Genetic deletion of *Ccr5* or *Cxcr6* abrogates the treatment-induced expansion of these subsets (expanded data for Fig. 3e. Statistical significance determined by unpaired Mann-Whitney tests.

Supplementary Figure 3.

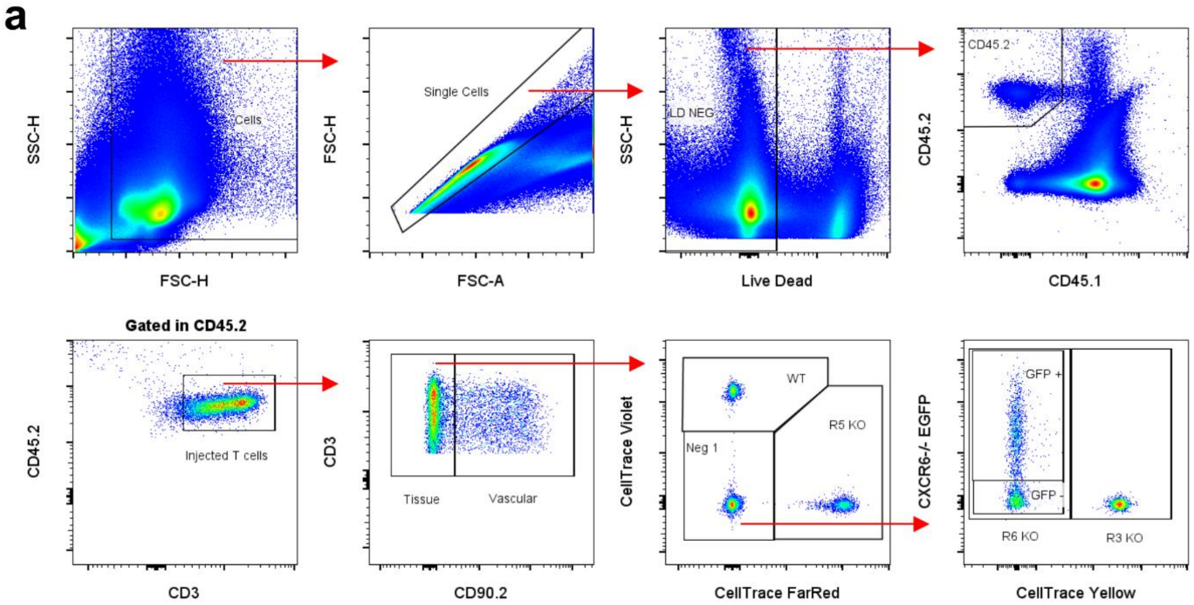

Supplementary Figure 3. Gating strategy for competitive homing assay. a. Flow cytometry gating strategy used to identify and track adoptively transferred T cells in recipient tissues 48 hours post-injection.

Supplementary Figure 4.

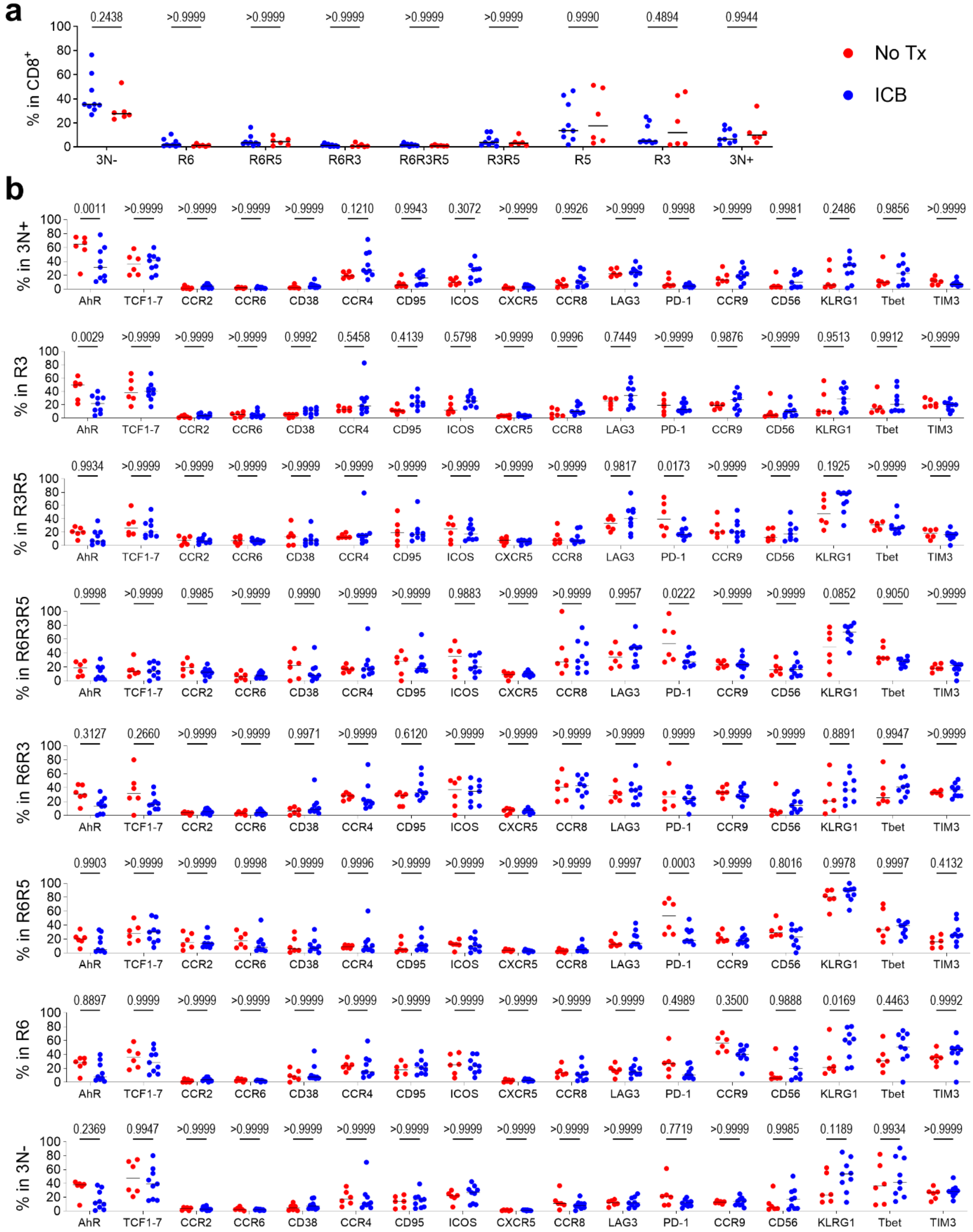

Supplementary Figure 4. Systemic impact of ICB on CR-defined subset frequency and phenotype. a. Percentages of CR-defined CD8$^+$ T-cell subsets in PBMCs from ICB-treated melanoma patients versus untreated (No Tx) controls (expanded data for Fig. 4b right). **b**. Expression of CRs, transcription factors, activation and exhaustion markers in circulating CR-defined CD8$^+$ T-cell subsets following ICB treatment (expanded data for Fig. 4c). Statistical significance determined by unpaired Mann-Whitney tests.
